# Speed and direction response profiles of neurons in macaque MT and MST show modest constraint line tuning

**DOI:** 10.3389/fnbeh.2013.00022

**Published:** 2013-04-04

**Authors:** Jacob Duijnhouwer, André J. Noest, Martin J. M. Lankheet, Albert V. van den Berg, Richard J. A. van Wezel

**Affiliations:** ^1^Center for Molecular and Behavioral Neuroscience, Rutgers UniversityNewark, NJ, USA; ^2^Department of Biophysics, Donders Institute for Brain, Cognition and Behaviour, Radboud UniversityNijmegen, Netherlands; ^3^Experimental Zoology Group, Department of Animal Sciences, Wageningen UniversityWageningen, Netherlands; ^4^Department of Cognitive Neuroscience, Donders Institute for Brain, Cognition and Behaviour, RUNMCNijmegen, Netherlands; ^5^Department of Biomedical Signals and Systems, MIRA, Institute for Biomedical Technology and Technical Medicine, Twente UniversityEnschede, Netherlands

**Keywords:** optic flow, visual motion, motion speed, motion direction, extrastriate cortex, area MT, area MST

## Abstract

Several models of heading detection during smooth pursuit rely on the assumption of local constraint line tuning to exist in large scale motion detection templates. A motion detector that exhibits pure constraint line tuning responds maximally to any 2D-velocity in the set of vectors that can be decomposed into the central, or classic, preferred velocity (the shortest vector that still yields the maximum response) and any vector orthogonal to that. To test this assumption, we measured the firing rates of isolated middle temporal (MT) and medial superior temporal (MST) neurons to random dot stimuli moving in a range of directions and speeds. We found that as a function of 2D velocity, the pooled responses were best fit with a 2D Gaussian profile with a factor of elongation, orthogonal to the central preferred velocity, of roughly 1.5 for MST and 1.7 for MT. This means that MT and MST cells are more sharply tuned for speed than they are for direction; and that they indeed show some level of constraint line tuning. However, we argue that the observed elongation is insufficient to achieve behavioral heading discrimination accuracy on the order of 1–2 degrees as reported before.

## Introduction

Optic flow is the visual motion pattern that emerges as one moves through the environment. The direction of self-motion, or heading, can be estimated from the optic flow field as it coincides with the direction of the point from which the visual scene expands in a radial pattern of motion (Gibson, 1950). However, heading estimation becomes more complex when the observer makes smooth pursuit eye movements while moving forward, as occurs for example when a stationary object in the scene is fixated on. Smooth pursuit eye movements result in coherent visual motion in the entire visual field that is superimposed on the optic flow. The result is a displacement of the focus of expansion with respect to the heading direction. To estimate heading from the flow fields present on the retina, the visual system needs to distinguish between the component of flow due to translational body movements and that due to rotational eye movements. This is known as the rotation problem.

Psychophysical studies have shown that human observers are able to compensate for pursuit during heading direction detection tasks. In these studies, subjects typically viewed large optic flow displays and indicated the perceived direction of their visually simulated heading. Even while making pursuit eye movements, the heading estimates were within a modest error range of 1–2 degrees (Warren and Hannon, 1990; van den Berg, 1993, 1996; Royden et al., 1994; Banks et al., 1996). In complementary experiments observers did not move their eyes, but were shown optic flow that included simulated eye rotations. Heading estimates in this case were less accurate, but observers showed systematic compensation for the added rotational component flow provided that the simulated scene contained depth information.

Several computational models have been put forward to explain the neurophysiological underpinnings of this behavioral ability (for reviews: Lappe, 2000; Britten, 2008). These models are typically based on the properties of neurons found in medial superior temporal (MST) area of the cerebral cortex. MST neurons respond selectively to visual optic flow patterns (Saito et al., 1986; Duffy and Wurtz, 1991, 1995) and are tuned to the vestibular components of self-motion (Page and Duffy, 2003; Gu et al., 2006; Angelaki et al., 2011). Interestingly, MST cell responses are modulated by efference copies of smooth pursuit eye movement signals, which suggest their importance in solving the rotation problem (Komatsu and Wurtz, 1988; Erickson and Thier, 1991; Bradley et al., 1996; Bremmer et al., 1997; Chukoskie and Movshon, 2009; Inaba et al., 2011). In addition, it has been suggested that MST partially compensates for pursuit based on the purely retinal signature of the pursuit on the optic flow field, i.e., the systematic distortions that occur when pursuit and heading are simulated with a flow field containing depth (Bremmer et al., 2010).

Area MST's main visual input comes from middle temporal (MT) neurons, which are tuned to unidirectional local motions (for a review: Born and Bradley, 2005). In one class of models, the template models, MST-like cells receive input from a mosaic of MT-like local motion detectors that corresponds to the flow that would arise from a particular heading (Perrone, 1992; Perrone and Stone, 1994, 1998; Crowell, 1997; Beintema and van den Berg, 1998; van den Berg and Beintema, 2000). The detected heading in these models corresponds to the position of the most active template-cell in a map of templates-cells tuned to different heading directions. A complication of this type of model is that an extremely large number of templates are required to cover the combinatorial explosion of heading parameters, eye rotations and scene layouts.

**Figure 1:**
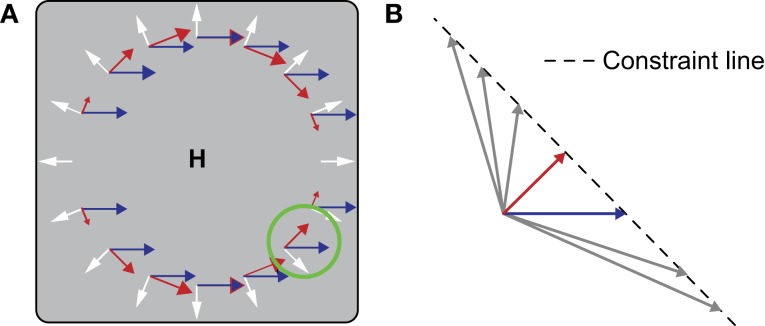
**(A)** During simultaneous (forward) locomotion and (leftward) smooth pursuit, the global flow pattern on the retina (gray square) is the vector sum (not shown) of a translational component (white) and a rotational component (blue). The center of the translational component coincides with the heading direction (*H*). To recover *H*, the velocity gain field model uses bi-circular motion templates (red arrows). Multiple templates cover the space of heading directions and pursuit speeds and directions. The template that is tuned to the present heading direction is invariant to the magnitude of the radial component and uniquely filters the rotational component. For this purpose a local constraint line tuning is used. **(B)** Magnification of the local tuning in the green circle. Responses are equal to all image velocities (arrows) that fall on the line of constraint (dashed line). The constraint line is perpendicular to the local direction of the bi-circular gradient. The advantage of using a combination of global bi-circularity and local constraint line tuning is that the same detector can be used for a large range of heading speeds, thus reducing the required number of templates.

In the velocity gain field model (Beintema and van den Berg, 1998), the combination space is reduced by the use of templates that uniquely pick up the rotational component of flow. The activity of these rotation templates is used to shift the peak of activity in a two-dimensional map of pure heading (i.e., without rotation) detectors. The output of the model, perceived heading, is the location of the peak in this map in a winner-takes-all fashion. The magnitude of the shift of the peak is modulated by an extra-retinal pursuit velocity signal (cf. Perrone and Krauzlis, 2008a), which is where the velocity gain field model derives its name from. The templates that pick up the rotational component of the optic flow field are structured such that all local flow preferences are perpendicular to those in the corresponding pure heading template. Because the heading templates have a radial structure, their complementary rotation templates have a bi-circular structure (red arrows in Figure 1A).

At the local level, motion velocities (i.e., the combination of speed and direction) are sampled in a special way in this model. At each given point in the bi-circular template, the sensitivity is equally strong to any velocity that falls on the line of constraint that runs orthogonal to the local preferred velocity (Figure 1B). As a result, the bi-circular detector is completely insensitive to the radial optic flow that the corresponding pure heading detector prefers, but does respond when a rotational component is added to the flow field. Note that because of this specific local motion tuning, which we call *constraint line tuning*, a bi-circular template perfectly matches the unidirectional flow resulting from a pursuit eye movement made parallel to the division of the two circular halves (blue arrows in Figure 1A). The advantage of templates built in this way is that they are invariant to the magnitude of the translational component of flow and scene layout. This invariance drastically reduces the number of templates required to solve the rotation problem. Similar local velocity tuning is used in Crowell's (1997) implementation of the Perrone and Stone (1994) template model.

The purpose of this study is to quantify local constraint line tuning in the primate visual system and determine if it's strong enough to support the velocity gain field model. If the bi-circular motion templates with local constraint-line tuning are present in MST, as postulated by the velocity gain field model, then stimulating the RF of these neurons with a small random dot stimulus moving in a range of speeds and directions should result in a ridge of activity that falls along the constraint-line in velocity space (v_x_ × v_y_). This tuning could be constructed from the projection of an ensemble of local motion detectors that occupy the same position in visual space and individually do not exhibit constraint line tuning.

Alternatively, the local motion detectors that provide the input to the bi-circular template cells could already be constraint-line tuned. Numerous studies have demonstrated that MT neurons are tuned to speed and direction, but speed tuning has typically only been measured in the preferred direction of the neuron (as determined at one speed) thus leaving unanswered whether perhaps the preferred speed increases at neighboring directions as required for constraint line tuning (Maunsell and van Essen, 1983; Felleman and Kaas, 1984; Lagae et al., 1993; Perrone and Thiele, 2001; Krekelberg et al., 2006). Two studies that did measure combinations of speed and direction did not find such an interaction using a moving bar stimulus (Rodman and Albright, 1987; Okamoto et al., 1999). However, an interaction between speed and direction consistent with constraint-line tuning was found in a few MT neurons when a moving dot stimulus was used (Okamoto et al., 1999). We tested for constraint line tuning in MT units by presenting the same drifting random dot stimuli used in MST that covered the entire MT receptive field.

## Methods

### Subjects

Two male Rhesus macaques were used in this study. All housing, handling, surgical, and recording procedures, as well as the experiments described in the paper, were approved by the Animal Use Committee (DEC) of Utrecht University, and were in agreement with national and international guidelines. To record from MT and MST neurons, the macaques were implanted with a head holding device placed centrally on the skull and a recording cylinder (Crist Instruments, Hagerstown, MD) that was placed over a craniotomy above the left occipital lobe.

### Single cell recordings

Extracellular single unit recordings were carried out using standard methods as described previously (Perge et al., 2005). In short, a parylene-insulated tungsten microelectrode (0.5–2 MΩ impedance at 1000 Hz; FHC Inc., Bowdoin, ME) was inserted through a needle that punctured the dura mater, and then brought into the proximity of a neuron using a servomechanism. The electrical signal recorded with the electrode was amplified (BAK Electronics Inc., Mt. Airy, MD), filtered at 24 dB/octave below 1000 Hz and above 2000 Hz, and digitized with a Micro1401 (CED, Cambridge, UK). These data were stored along with synchronization pulses from the stimulus computer for offline analysis. In parallel, we loosely isolated the action potentials with a window discriminator (BAK Electronics Inc., Mt. Airy, MD) for the purpose of online analysis.

Cortical areas MT and MST were identified by the recording position and depth, the transition between gray matter, white matter, and sulci along the electrode track, and by functional properties. For MT, these were the prevalence of direction-selective units, the receptive field size according to eccentricity, and the change of direction tuning along the electrode penetration. Area MST was functionally identified by the prevalence motion sensitive neurons that had large receptive fields that could overlap the fixation point and extend well into the ipsilateral visual hemifield.

### Stimuli

Visual stimuli were generated with OpenGL on an Apple PowerMac G4 (1.25 GHz) and back projected (JVC DLA-S10) onto a translucent screen with a resolution of 1152 × 786 pixels at 75 Hz refresh rate. The display subtended 105° × 72° at a viewing distance of 58 cm.

During the experiment, the monkey was motivated by liquid rewards to keep his gaze within a 1° radius from a red dot at the center of screen. The rewards were given at 500–1000 ms intervals, independently of the visual stimulation.

Full field optic flow stimuli, which drive MST and MT cells well, were presented while searching for a neuron by slowly lowering an electrode into the cortex. In short, these screen filling neuron-search stimuli consisted of 756 white (46 cd/m^2^) anti-aliased dots on a dark (0.11 cd/m^2^) background with a diameter of 3 pixels, corresponding to 0.27° foveally. The dots moved as if the observer rotated around or translated along an axis oriented in one of a range of directions. This range was, in polar coordinates centered on the fixation point, 0–315° in steps of 45° by 0°, 30°, or, 90° eccentricity. Rotation speed was ±120°/s, translation speed was ±12 ms^−1^ through a cloud of dots ranging from 38 cm to 10 m in front of the monkey. Trials lasted 333 ms and were presented in random order within a block (set of all rotation, speed, direction combinations). For efficiency, the trials were presented without a blank interval. Dot positions were refreshed at the beginning of each presentation.

Once a responsive neuron was isolated by positioning the electrode in close proximity, the extent of the receptive field was estimated manually by moving a slit of light across the visual field and listening to the neuron's response which was made audible in headphones. In case an MT neuron was isolated, the constraint line stimulus was positioned congruently with the estimated receptive field location. Otherwise, if an MST cell was being recorded, the stimulus was placed on a responsive subregion of the receptive field. The diameter of the constraint line stimulus in this case was typically chosen to be 0.76 times the eccentricity of its center plus a 4.6° constant, corresponding to the average MT receptive field size at that location (Raiguel et al., 1995).

**Figure 2:**
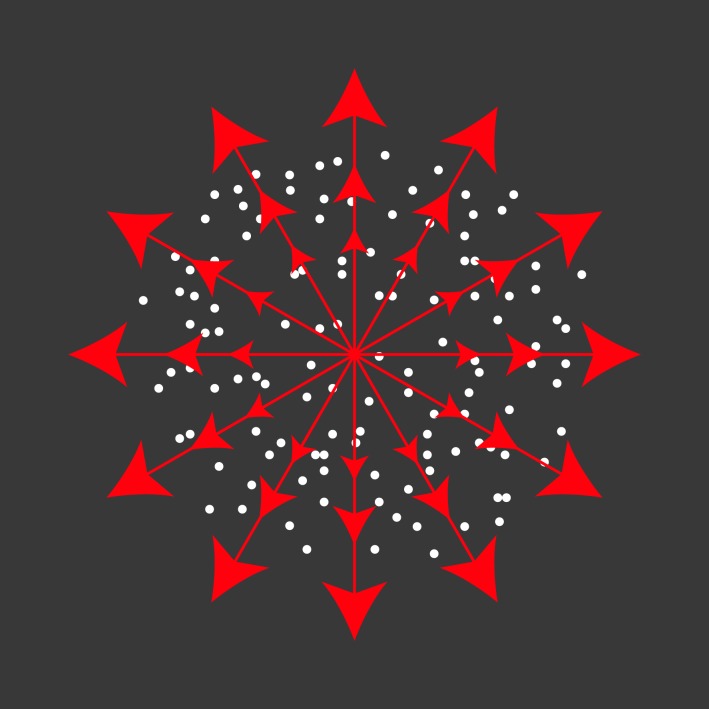
**The constraint line stimulus consisted of light dots inside a circular aperture on a dark background.** Per trial, the dots moved within the aperture in one of 12 or 8 evenly spaced directions and at one of a range of at least five different speeds. The stimulus was positioned concentrically with the receptive field of an MT cell or on a responsive area within an MST receptive field.

The constraint line stimulus (Figure 2) consisted of a circular patch of dots with a density of half a dot per square degree. Luminance and other dot parameters were identical to the search stimulus. The dots moved within the patch in any of 12 or 8 directions and at different speeds. The number of directions was reduced from 12 to 8 when about one third of the first monkey's data was collected. The usual speed range at the start of a recording was 0, 16, and 64°/s. Speeds centered on the preferred speed as determined by automated online analysis were added to this list as the experiment ran. Speed 0°/s in one nominal direction was always present in the speed settings range. The stimulus set of all data presented in this paper contained at least six different speeds including speed 0°/s. Similar to the unit-search experiment, trials were presented in random order within a block, dot positions were refreshed at the beginning of a trial, and no blank was presented between trials. The stimulus presentation duration was 333 ms. The stimulus was programmed such that the dots moved at a constant speed over the surface of a virtual sphere centered on the cyclopean eye of the monkey. This prevented the perspective distortion of the aperture and dot velocity gradients that would be apparent if the dot speed was constant and the aperture circular on the screen, especially at large eccentricities.

### Analysis

We isolated single and multi units from our analog electrode data using Spike2 version 5.12 (CED, Cambridge, UK). Next, we determined the response of each unit to the stimuli by determining the number of spikes occurring within the time window of each presentation shifted by an estimate of the latency of the unit. The latency was determined as follows. We slid a 333 ms long time window from 15 to 150 ms after the appearance of the stimulus on the screen with increments of 1 ms, and smoothed the resulting temporal-offset vs. firing rate function with a 15 ms boxcar filter. We defined the latency as the temporal-offset at which the responses across the trials showed the largest variance. We reasoned that a large variance corresponds to the strongest velocity tuning. A similar method was employed by Smith et al. (2005).

**Figure 3:**
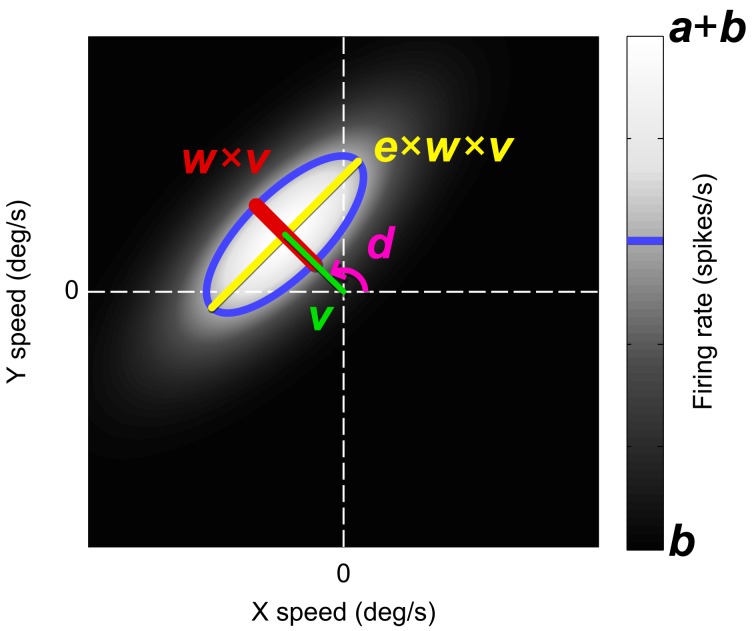
**The six-parameter model used to fit the neural responses (grayscale) to the horizontal (X) and vertical (Y) stimulus speeds.** The model was a 2D Gaussian in velocity space with its center at the polar coordinates *d* (the preferred direction) and *v* (the preferred speed). The width of the Gaussian in the preferred direction, *w*, was expressed as a Weber fraction of *v*. The Gaussian width in the orthogonal direction was defined as the product of *e*, *w*, and *v*. Larger *e* means more constraint line tuning. Parameter *b* was an offset representing the baseline firing rate and *a* was the amplitude of the Gaussian. The blue contour line indicates one standard deviation from the Gaussian center.

Thus, we obtained for each unit a data-set with three values per trial, i.e., the stimulus displacement in degrees per second in the horizontal (*x*) and the vertical direction (*y*), and the observed firing rate (*r*). We fitted these data with a six-parameter model that centers a 2D Gaussian on the preferred speed and direction of the neuron (Figure 3). This model is defined in the domain (*X****′***, *Y****′***) which is the velocity space (*X, Y*) rotated such that the *x*-axis aligns with the estimated preferred direction (*d*) of the unit:

$$\begin{array}{l} X^{\prime }=\cos d\;X+\sin d\;Y\\ Y^{\prime }=\text{-}\sin d\;X+\cos d\;Y \end{array}$$

We report *d* in units of degrees in the remainder of this paper. We obtain the tuning profile

$$P=\text{G}\left(X^{\prime },v,wv\right)\text{ G}(Y^{\prime },0,ewv)\;$$

as the product of two standard Gaussians functions G (*x*, μ, σ) where *v* represents the preferred speed of the neuron in degrees per second, *w* represents the width of the 2D Gaussian parallel to the preferred direction expressed as a fraction of *v* (Weber, 1846), and *e* represents the elongation of the 2D Gaussian perpendicular to the preferred direction. Finally, we obtain the firing rate estimate *R* in spikes per second by normalization and scaling of *P*,

$$R=a\left(\frac{P-P_{min}}{P_{max}-P_{min}}\right)+b$$

where *b* represents the baseline firing rate and *a* the amplitude of the 2D Gaussian (excluding *b*), both in spikes per second.

We used Matlab's (The Mathworks, Inc.) lsqcurvefit function (trust-region-reflective algorithm) to find values and 95% confidence limits for these coefficients that best fitted the unbinned data (one datum per trial) in the least-squares sense. The preferred speed parameter *v* was constrained between 0 and 512°/s, the scalar *w* between 0.01 and 50, the scalar *e* between 0.01 and 1000. The scaling parameters *a* and *b* were constrained between 0 and the maximum observed firing rate. The circular preferred direction parameter *d* was practically unbound. In this study, we focus primarily on the parameter *e* as it reflects the amount of constraint line tuning. Values larger than one indicate that the unit is responsive to a set of velocities that fall on the line of constraint whereas values of one and lower indicate that the unit is not constraint line tuned.

## Results

**Figure 4:**
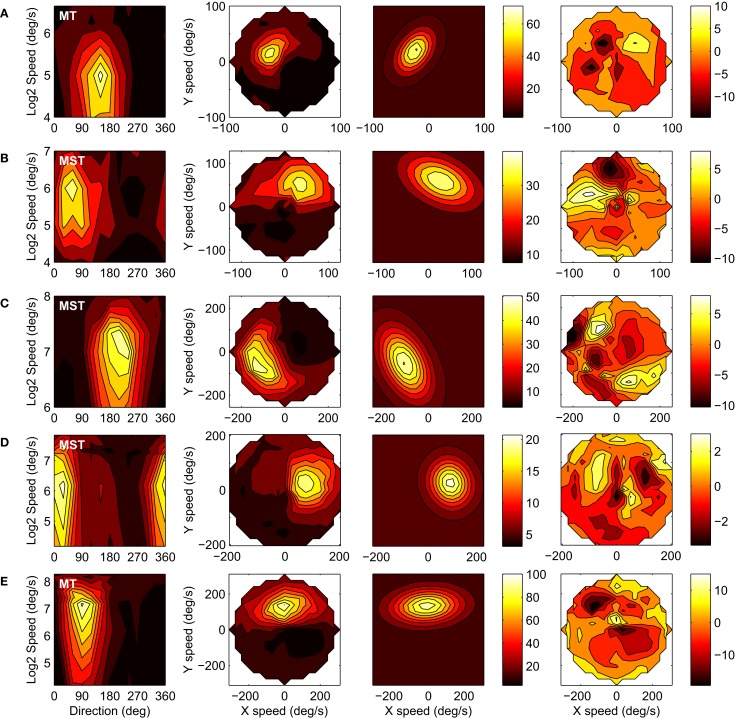
**The velocity tuning of five example units (rows).** The columns from left to right show: (1) The response in spikes per second (color scale) as a function of stimulus direction and stimulus speed; (2) The same data as a function of the horizontal (X) and vertical (Y) components of stimulus speed, i.e., velocity space; (3) Six-parameter model fits to the velocity space data; (4) Residuals of the model fits. **(A)** Monkey-A MT unit: *r*^2^ = 0.93, *d* = 144, *v* = 31, *w* = 0.55, *e* = 1.6, *a* = 63, *b* = 8. **(B)** Monkey-A MST unit: *r*^2^ = 0.73, *d* = 65, *v* = 66, *w* = 0.47, *e* = 1.8, *a* = 29, *b* = 11. **(C)** Monkey-F MST unit: *r*^2^ = 0.89, *d* = 207, *v* = 121, *w* = 0.51, *e* = 1.8, *a* = 42, *b* = 8. **(D)** Monkey-F MST unit: *r*^2^ = 0.83, *d* = 19, *v* = 97, *w* = 0.56, *e* = 1.2, *a* = 16, *b* = 5. **(E)** Monkey-F MT unit: *r*^2^ = 0.88, *d* = 94, *v* = 132, *w* = 0.47, *e* = 2.0, *a* = 83, *b* = 12.

We successfully recorded the velocity tuning of 42 MST and 23 MT units in Monkey A, and 52 MST and 21 MT units in Monkey F. A recording was considered successful when, after fitting the six-parameter model, the 95% confidence interval of the response amplitude parameter *a* did not include zero. Figure 4 shows the responses of five representative units to our velocity stimuli. The four columns from left to right show: (1) The response in spikes per second (color scale) as a function of stimulus direction and stimulus speed; (2) The same data as a function of the horizontal (X) and vertical (Y) components of stimulus speed, i.e., velocity space; (3) The six-parameter model fits to the velocity space data; (4) The residuals of the model fits. Per row, the same color scale limits are used in all panels except the residuals plot. For the purpose of plotting the response profiles in the three rightmost panels in each row we binned the responses elicited by the speed × motion direction combinations in a rectangular 19 × 19 grid in velocity space. The fits captured the data reasonably well, as evidenced by the mean *r*^2^ value of 0.66 (*SD* = 0.15) across the 138 cells in our sample and the general lack of structure in the residuals. A notable systematic deviation from the fits is that the measured response profiles of some units curved on a circle of equal speed in velocity space, whereas the fit profiles were by definition straight perpendicular to the axis of preferred direction. This curvature is especially clear in row C and in the additional high-*e* examples in Figure 8 (see Discussion).

**Figure 5:**
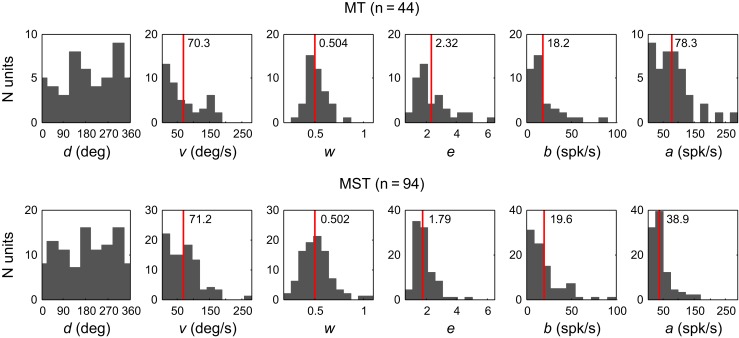
**Histograms of the fit estimates of the six-parameter model across the population of MT units (Top) and MST units (Bottom).** Red vertical lines indicate the means.

Figure 5 shows the distributions of the fit estimates of the six-parameter model across the population of MT units (top) and MST units (bottom). The numbers in the panels and the red vertical lines indicate the mean values for each parameter except preferred direction *d* because it is periodic. We used the two-sided Wilcoxon rank sum test to see if the corresponding parameter estimates for MT and MST stem from identical continuous distributions with equal medians. This is very unlikely for the response amplitude parameter *a* (*p* < 0.001) and the elongation parameter *e* (*p* = 0.009). No significant difference was observed between the other parameters. Applying *t*-tests on the parameter distributions after logarithmic transformation to better approximate normality yielded similar results. Mean *a* was higher in MT (78 spikes/s, *SD* = 57) than in MST (39 spikes/s, *SD* = 32). This makes sense because the MT units were driven with a stimulus that typically covered the entire receptive field, whereas only a portion of the MST receptive fields was stimulated. Mean *e* was slightly larger in MT (2.32, *SD* = 1.15) compared to MST (1.79, *SD* = 0.64).

**Figure 6:**
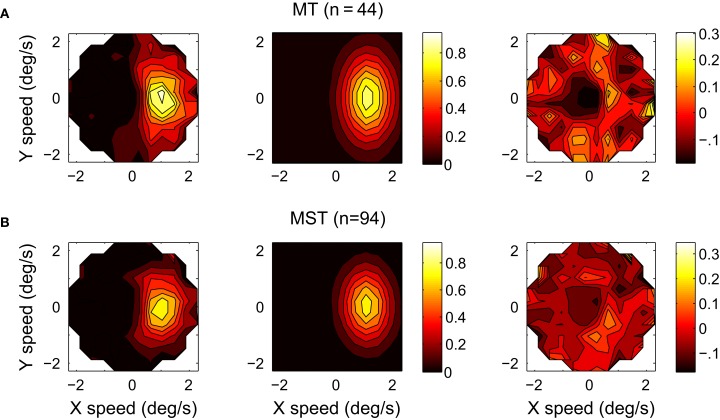
**The response profiles of the normalized and pooled populations of 44 MT (A) and 94 MST (B) units recorded in two monkeys.** Columns from left to right: The response profile in velocity space; The six-parameter model fits; The residuals (MT: *r*^2^ = 0.80; MST: *r*^2^ = 0.82).

To further investigate the tuning of the MT and MST populations, we calculated the normalized and pooled velocity response profiles of the 44 MT and 94 MST units. The measured response profiles of the individual units were normalized by fitting the six-parameter model and then aligning the preferred direction *d* with zero, dividing the stimulus speeds by *v*, subtracting *b* from the firing rate and dividing the remainder by *a*. Figure 6 shows the profiles of the median MT and MST population responses as a function of velocity space (12 × 12 bins) truncated at normalized speed 2.5. We fitted the six-parameter model to the means of the median firing rates across trials of each unit at the location of the bins. Not every unit had a response at each bin, this occurred for example when the fastest speed presented to a unit was less than 2.5 its preferred speed. The fit to the MT population yielded an *r*^2^ of 0.80 and the following estimates with 95% confidence intervals: *d* = −0.07 ± 4.60, *v* = 1.11 ± 0.07, *w* = 0.56 ± 0.08, *e* = 1.70 ± 0.26, *a* = 0.84 ± 0.09, *b* = 0.02 ± 0.03. The fit to the MST population had an *r*^2^ of 0.82 and *d* = −2.61 ± 4.26, *v* = 1.07 ± 0.06, *w* = 0.56 ± 0.07, *e* = 1.52 ± 0.22, *a* = 0.76 ± 0.08, *b* = 0.01 ± 0.02. These estimates of *e* are slightly lower than the mean of the *e* values obtained by fitting the response profiles of all individual MT (*e* = 2.32, *SD* = 1.15) and MST units (*e* = 1.96, *SD* = 0.87). This reduction may be a result of binning the responses in a rectangular grid in the normalized and pooled analysis as opposed to using the speed × direction data points in the individual fits which occupy concentric circles. Because the low speed data points are more tightly clustered than the high speed data points, they end up in a smaller number of rectangular bins. This means that they have a lower relative weight in the fit.

The 95% percent confidence intervals on the estimates for *e* in the pooled MT and MST data comfortably exclude one, showing that the elongation parameter plays a meaningful role in explaining the variance of the data. To further investigate the necessity of *e* in the six-parameter model we performed alternative fits with a five-parameter model from which *e* was removed by fixing it at unity. We used a likelihood ratio test to compare the residual sum of squares of the full and reduced models while correcting for the different number of parameters. The inclusion of *e* resulted in significantly better fits for both the pooled normalized data of MT [*F*_(1, 366)_ = 135.3, *p* < 0.001] and MST [*F*_(1, 378)_ = 102.8, *p* < 0.001].

The preferred speed *v* in MT had a of mean 70.3°/s (*SD* = 55.1) which is higher than the mean of around 32°/s reported previously (Maunsell and van Essen, 1983; Rodman and Albright, 1987). This may be caused by our use of Gaussian fits to the linear stimulus speed instead of to the log-speed as used in many other studies (e.g., Priebe et al., 2003; Krekelberg et al., 2006). Gaussian fits (linear or logarithmic) are not very good at capturing the sharply peaked tuning that some MT neurons are known to exhibit, which can also lead to overestimation (Perrone, 2006). In our sample, MST had similar preferred speeds to MT (mean = 71.2°/s, *SD* = 48.5).

In the fit model, the width of the speed tuning is described with *w*, a Weber fraction of the magnitude of the preferred speed. Therefore, the tuning width of speed in deg/s is the product of *w* and *v*. The mean width of the speed tuning was 33.5°/s (*SD* = 24.5) in MT and 34.8°/s (*SD* = 22.3) in MST. The tuning to stimulus direction in degrees is 2 arctan(*ew*). We found a mean directional tuning width in MT of 91.2° (*SD* = 25.8), which agrees well with previous estimates (e.g., Maunsell and van Essen, 1983; Albright, 1984). In MST, the directional width tuning was 78.9° (*SD* = 17.3); sharper than in MT [*t*-test, *t*_(136)_ = 3.30, *p* = 0.0012].

## Discussion

The velocity gain field model (Beintema and van den Berg, 1998) employs large field motion detectors that combine a global bi-circular layout (Figure 1A) with local constraint line tuning (Figure 1B). Because of these properties, the detectors are able to pick up the visual effects of eye rotation while at the same time be invariant to the radial component of optic flow (i.e., heading speed). In the velocity gain field model, the constraint line tuning was complete, i.e., the local velocity tuning was infinitely extended in the direction perpendicular to the local gradient of the bi-circular field. Or, in terms of the six-parameter model fit that we used in this study, the elongation parameter *e* was infinite.

In this study we sought to determine *e* experimentally. By pooling the normalized data of all MT units and then fitting the six-parameter model (Figure 6) we found an *e*-value of 1.70 with a 95% confidence interval of ±0.26. For the MST population we found an *e* of 1.52 ± 0.22.

Plotting the responses as a function of horizontal and vertical velocity instead of the more conventional direction and speed diagrams makes the relative sensitivity of the neuron to changes in stimulus speed and direction more apparent. In the conventional plots (e.g., left column Figure 4), it is hard to compare the influence of a change in direction on the firing rate (spikes/s per degree around a visible direction) with the influence of a change in speed on the firing rate (spikes/s per degree of visible angle/s) because they are in entirely different units. However, from the horizontal and vertical velocity diagrams in Figure 6 we can conclude that MT and MST units are more sharply tuned to speed than they are to direction, i.e., the gradient of the response (in spikes/s per deg/s) is stronger over the line through the preferred velocity and the origin than over the circle of equal speeds containing the preferred velocity.

**Figure 7:**
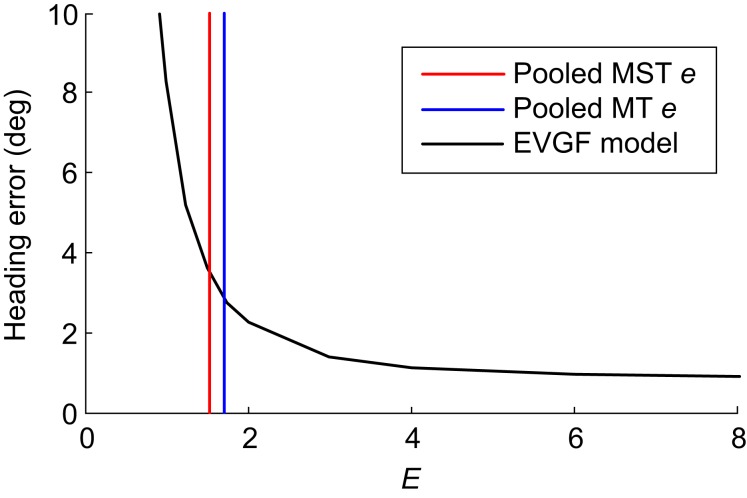
**Heading error (i.e., under-compensation for pursuit) in the elaborated velocity gain field (EVGF) model as a function of *E*, the amount of local constraint line tuning.** Pursuit-compensation starts to saturate at a realistic level from *E* ≈ 3. The vertical lines indicate the values of *e* observed in our pooled MT and MST samples. This analysis suggests that *e* is not sufficient to support constraint line tuning as proposed in the velocity gain field model.

It is important for the purpose of the present study to estimate whether the amount of elongation *e* that we found is sufficient for the velocity gain field model to achieve a realistic level of pursuit compensation. To this end, we elaborated the velocity gain field model by implementing a Gaussian weighting kernel along the line of constraint of the local velocity tuning of which the width was controlled by a parameter *E*, similar to the parameter *e* used in the six-parameter model fit. We simulated a 0.5 m/s heading through a cloud of dots (depth range 0.5–3.73 m) while making 5°/s pursuit eye movements. A similar heading situation has been used in a psychophysical study (Royden et al., 1992). Figure 7 shows the relation between *E* and the heading error (under-compensation for real pursuit) predicted by the elaborated velocity gain field model. Settings for the other parameters in the velocity gain field model were (parameter names as in Beintema and van den Berg, 1998): Expansion template *O*_0_ : (1, 0, 0), i.e., preferred heading direction straight ahead; Rotation+Translation templates *O*_*z*_ : (0, 0, *R*); *O*_*−z*_ : (0, 0, −*R*); preferred rotation speed of the templates *R* = 5°/s. The difference of template activities η (O_z_−O_−z_)/(2R) is the gain-field term that compensates for the effect of the eye's rotation speed η°/s. The templates implemented motion parallax scatter weighting through a function with σ_*s*_ = 2 (Beintema and van den Berg, 1998; their section 5).

We found that the heading error drops exponentially with increasing *E*, and starts to level off at values of *E* >3. This simulation shows that infinite *E*, as assumed in the original velocity gain field model is not necessary for robust pursuit compensation. However, it also demonstrates that the values of *e* observed in the pooled MT and MST units (vertical lines in Figure 7) provide insufficient constraint line tuning to reduce heading errors by the model sufficiently to match results from psychophysical studies. Thus, neither the tuning properties of MT neurons or the tuning properties of sub regions of MST neurons match the constraint line properties assumed in the gain field model.

**Figure 8:**
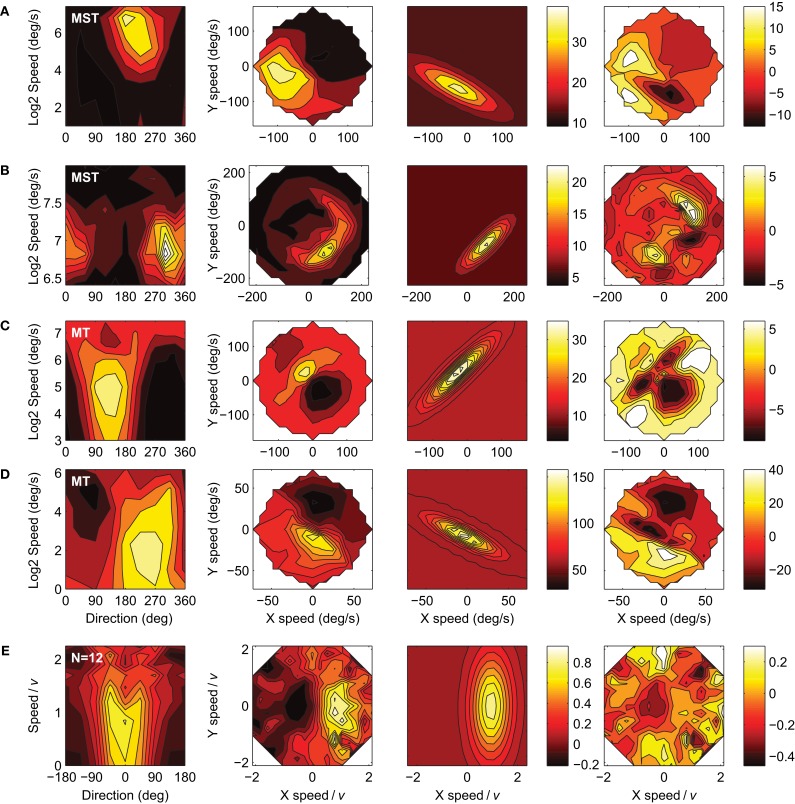
**Four examples of the 12 units with *e* values greater than 3 (first four rows) and the pooled and normalized average of those 12 neurons (bottom row).** Columns represent the data, fits and residuals as in Figure 4. The response profiles in **(C)**, **(D)**, and **(E)** show slight bifurcations at high speed values in the speed-direction domain (left panels), as is expected with high values of *e*. **(A)** Monkey-A MST unit: *r*^2^ = 0.71, *d* = 242, *v* = 68, *w* = 0.34, *e* = 3.76 ± 0.80, *a* = 25, *b* = 13. **(B)** Monkey-F MST unit: *r*^2^ = 0.65, *d* = 315, *v* = 112, *w* = 0.22, *e* = 3.43 ± 1.03, *a* = 16, *b* = 6. **(C)** Monkey-F MT unit: *r*^2^ = 0.52, *d* = 134, *v* = 35.9, *w* = 0.50, *e* = 4.57 ± 1.62, *a* = 23, *b* = 12. **(D)** Monkey-A MT unit: *r*^2^ = 0.71, *d* = 243, *v* = 8.43, *w* = 0.80, *e* = 4.38 ± 2.55, *a* = 98, *b* = 60. **(E)** Pooled and normalized population mean of the 12 units. *r*^2^ = 0.58, *d* = −0.53 ± 4.78, *v* = 0.95 ± 0.07, *w* = 0.58 ± 0.10, *a* = 0.81 ± 0.11, *b* = 0.05 ± 0.04, and *e* = 2.55 ± 0.57. Error ranges are 95% confidence intervals.

A mechanism as proposed in the velocity gain field model may operate on the subset of units that have an *e* >3. However, there are two reasons why we believe our data does not support this. First, we found that 21% of our MT sample met this criterion, compared to only 3% of our MST sample. Examples of these responses are shown in Figure 8. The higher fraction of high *e* (and higher mean *e*) in MT seems incompatible with an interpretation of large *e* serving a role in pursuit compensation by means of MST-like large scale motion templates. Second, the velocity tuning profiles with the highest *e* do not *necessarily* correspond to constraint line tuning. The response profiles often show a curvature along lines of equal absolute speed. This is reminiscent of the velocity tuning observed in so called S2 type MT cells that have a trough in the velocity tuning profile in the anti-preferred velocity (i.e., inhibition of response by stimulus with opposite direction but equal absolute speed of the most excitatory stimulus; Rodman and Albright, 1987). Moreover, curvature of the response profile in velocity space is predicted by a model that proposes that MT neurons contain subunits that are tuned to the primary motion direction at a certain speed and off-axis directions with progressively lower preferred speeds (Perrone, 2004; Perrone and Krauzlis, 2008b). In line with this, one would expect curvature in MST when a range of preferred directions is present under the subfield stimulus, for example, for stimuli near the focus of an RF tuned to radial optic flow. The fit-model used in the present study does not have the explicit ability to capture this curvature, and instead seems to have attempted to cover it by inflating *e*, resulting in some relatively poor fits in Figure 8. It is an open question at this point to what extent the performance of the velocity gain field model would be impacted from curvature of the constraint line.

A number of other studies have reported on striate and extrastriate cortical neurons that show velocity tuning to moving random dots that is compatible with high values of *e*. Okamoto et al. (1999) proposed a model of pattern-motion detection (Movshon et al., 1985) by MT neurons that predicted that, when stimulated with a moving dot, pattern cells show a unimodal direction tuning over the entire range of speeds, but component cells show a transition from unimodal direction tuning at low speeds to bimodal direction tuning at high speed, corresponding to a high *e* in our terminology. Six MT cells out of 15 that were classified as component cells (out of a total sample of 35 MT neurons) corroborated this prediction. Although specifics of the Okamoto et al. (1999) model have been disputed (Perrone and Krauzlis, 2008b) their data strongly suggest that neurons with high *e* are component cells. Our paradigm lacked an independent measure of pattern-component selectivity. However, it has been reported that MST neurons are mostly pattern selective (60%) and very rarely component selective (9%), whereas in MT, roughly equal portions of pattern selective, component selective and unclassifiable units are found (Movshon et al., 1985; Khawaja et al., 2009). This finding might explain why we found a higher incidence of high *e* values in MT than in MST. The bifurcations of the MT velocity tuning profiles in the Okamoto et al. (1999) study are more dramatic than even our strongest examples (Figure 8). The most obvious differences between that study and the present one are the use of stimuli consisting of a single dot and the use of anesthetized animals. Interestingly, it has been shown that anesthesia can increase the number of component selective responses and decrease the number of pattern selective responses in MT (Pack et al., 2001). Very clear bifurcations at high speeds have also been demonstrated in neurons located in cat areas 17 and 18 when stimulated with moving random dot pattern (Hammond and Reck, 1980; Crook, 1990; Skottun et al., 1994). Thus, taking our data and the literature together, the view emerges that high *e* values become rarer going down the visual stream from V1 to MT component to MT pattern to MST neurons, contrary to what would be expected if pursuit compensation in heading detection operates as proposed in the velocity gain field model.

Some studies show that unlike MT neurons, which are tuned to a specific speed, i.e., their response drops for speeds slower and faster than the preferred speed, the firing rate of many MST cells increases sigmoidally with stimulus speed (Tanaka and Saito, 1989; Inaba and Kawano, 2010) and recently a model has been proposed that explains this transition from speed tuning to speed coding (Perrone, 2012). In the present study, we found no significant differences between the preferred speeds *v* in MT and MST or between the speed tuning widths *w* in these areas. Tanaka and Saito (1989) used complex flow stimuli (e.g., radial expansion) with a limited speed range. Inaba and Kawano (2010) employed, like us, laminar flow stimuli with a large range of speeds. One salient difference with the present study is the use of large (80 × 80°) stimuli. We found MST peak firing rates of on average 39 spikes/s whereas the MST neurons in Inaba and Kawano (2010) saturated at 100–150 spikes/s. If the sigmoidal saturation of the speed response curve is somehow dependent on the total activity of the neuron, then the low output generated by the small patches used in our study could have led to the speed tuning apparent in our data. Another difference is that Inaba and Kawano (2010) used continuous motion created by physically drifting the projection of a random dot pattern whereas we used apparent motion. We increased stimulus speed by increasing the frame to frame displacements of the dots, meaning that the fraction of correlated dot pairs was reduced at high speeds. The relatively small stimuli and low refresh rate of our projector (75 Hz) contributed to this effect. Orban et al. (1995) using 100 Hz apparent motion stimuli of typically 20° × 20° also found (broad) speed tuning in MST, with reduced firing rates at high speeds.

In conclusion, we found a significant elongation of the velocity tuning profiles along the axis orthogonal to the preferred motion direction of MT and MST cells. This means that MT and MST cells are more sharply tuned to speed than to direction. However, analysis of an elaboration of the velocity gain field model presented in this study suggests that the observed elongation is too modest to constitute sufficient local constraint line tuning to support the velocity gain field model.

### Conflict of interest statement

The authors declare that the research was conducted in the absence of any commercial or financial relationships that could be construed as a potential conflict of interest.
